# Levels of Periplasmic Nitrate Reductase during Denitrification are Lower in *Bradyrhizobium japonicum* than in *Bradyrhizobium diazoefficiens*

**DOI:** 10.1264/jsme2.ME19129

**Published:** 2020-06-16

**Authors:** Arthur Fernandes Siqueira, Masayuki Sugawara, Haruka Arashida, Kiwamu Minamisawa, Cristina Sánchez

**Affiliations:** 1 Graduate School of Life Sciences, Tohoku University, 2–1–1 Katahira, Aoba-ku, Sendai 980–8577, Japan

**Keywords:** *Bradyrhizobium*, *cbb*_3_ oxidase, competitive growth, denitrification, periplasmic nitrate reductase

## Abstract

Soybean plants host endosymbiotic dinitrogen (N_2_)-fixing bacteria from the genus *Bradyrhizobium*. Under oxygen-limiting conditions, *Bradyrhizobium diazoefficiens* and *Bradyrhizobium japonicum* perform denitrification by sequentially reducing nitrate (NO_3_^–^) to nitrous oxide (N_2_O) or N_2_. The anaerobic reduction of NO_3_^–^ to N_2_O was previously shown to be lower in *B. japonicum* than in *B. diazoefficiens* due to impaired periplasmic nitrate reductase (Nap) activity in *B. japonicum*. We herein demonstrated that impaired Nap activity in *B. japonicum* was due to low Nap protein levels, which may be related to a decline in the production of FixP and FixO proteins by the *cbb*_3_-type oxidase.

Soybean is a globally important leguminous crop that generally hosts endosymbiotic dinitrogen (N_2_)-fixing bacteria from the genus *Bradyrhizobium* (Argaw, 2014). Under oxygen-limiting conditions, some species of soybean-associated *Bradyrhizobium* may perform denitrification as an alternative respiratory process and sequentially reduce nitrate (NO_3_^–^) or nitrite (NO_2_^–^) to nitric oxide (NO), nitrous oxide (N_2_O), and N_2_ gases. Denitrification is functional in free-living bradyrhizobia at the soybean rhizosphere and in symbiotic bradyrhizobia inside the root nodules (Mesa *et al.*, 2004; Sameshima-Saito *et al.*, 2006b; Sánchez *et al.*, 2011; Inaba *et al.*, 2012).

*Bradyrhizobium diazoefficiens* (reclassified from *Bradyrhizobium japonicum* [Delamuta *et al.*, 2013]; type strain USDA 110) may completely reduce NO_3_^–^ to N_2_. These reactions depend on *napEDABC*, which encodes periplasmic NO_3_^–^ reductase (Nap); *nirK*, which encodes copper-containing NO_2_^–^ reductase (NirK); *norCBQD*, which encodes *c*-type NO reductase (Nor); and *nosRZDYFLX*, which encodes N_2_O reductase (Nos) (Sánchez *et al.*, 2011). While *B. diazoefficiens* is a complete denitrifier, *B. japonicum* (type strain USDA 6) lacks the *nos* gene cluster and is, thus, unable to reduce N_2_O to N_2_ (Sameshima-Saito *et al.*, 2006b; Itakura *et al.*, 2009; Kaneko *et al.*, 2011; Siqueira *et al.*, 2014).

We previously screened the growth of 11 strains of *B. japonicum* and 15 strains of *B. diazoefficiens* and found that anaerobic (≈0% O_2_) growth by *B. japonicum* with NO_3_^–^ as the electron acceptor was significantly lower than that by *B. diazoefficiens*; however, no significant differences were observed between the growth rates of *B. japonicum* and *B. diazoefficiens* strains under microaerobic (≈2% O_2_) and aerobic (≈12% O_2_) growth conditions in the presence of NO_3_^–^ (Siqueira *et al.*, 2017). The lower growth rate of *B. japonicum* in anaerobiosis was not explained by the absence of *nos*, but by markedly impaired Nap activity that had a negative impact on the reduction of NO_3_^–^ to N_2_O (Siqueira *et al.*, 2017). *B. japonicum* conserved the FixLJ–FixK_2_ regulatory cascade, which mediates the response of the *nap* operon to low oxygen and NO_3_^–^; the binding sites for FixK/FNR (fumarate and nitrate reductase) regulators upstream of *napE* were also conserved (Robles *et al.*, 2006; Bueno *et al.*, 2017; Siqueira *et al.*, 2017). Additionally, *napA* transcript levels were similar in *B. japonicum* and *B. diazoefficiens* (Siqueira *et al.*, 2017). Thus, we proposed that impaired Nap activity in *B. japonicum* may be due to posttranscriptional events (Siqueira *et al.*, 2017).

In the present study, we used the type strains *B. diazoefficiens* USDA 110 (Bd-USDA 110) and *B. japonicum* USDA 6 (Bj-USDA 6) to expand on our earlier research on the mechanisms responsible for impaired Nap activity in *B. japonicum* under denitrifying conditions.

*Bradyrhizobium* cells were precultured at 30°C in HM salt medium (Cole and Elkan, 1973) supplemented with 0.1% L-(+)-arabinose and 0.25% (w/v) yeast extract. HM medium supplemented with trace metals (Sameshima-Saito *et al.*, 2006a) and 10‍ ‍mM KNO_3_ (HMMN medium) was used in all assays. *Escherichia coli* cells were grown at 37°C in Luria–Bertani medium (Miller, 1972). The following antibiotics were used for the *Bradyrhizobium* culture: tetracycline (Tc, 100‍ ‍μg mL^–1^), spectinomycin (Sp, 100‍ ‍μg mL^–1^), streptomycin (Sm, 100‍ ‍μg mL^–1^), and polymyxin B (50‍ ‍μg mL^–1^). Tc (50‍ ‍μg mL^–1^), Sp (50‍ ‍μg mL^–1^), and Sm (50‍ ‍μg mL^–1^) were used for the *E. coli* culture.

In growth experiments, precultured cells were inoculated into 34-mL test tubes containing 5‍ ‍mL of HMMN medium. The initial optical density at 660 nm (OD_660_) was adjusted to 0.01. In the competition experiment, initial cell numbers were adjusted to 3×10^7^‍ ‍cells‍ ‍mL^–1^ and cells were used at a 1:1 ratio. Aerobic (≈12% O_2_), microaerobic (≈2% O_2_), and anaerobic (≈0% O_2_) treatments were prepared and monitored as previously described (Siqueira *et al.*, 2017). Cells were grown at 30°C with reciprocal shaking at 300 rpm. Growth was monitored daily by measuring the OD_660_ of the cultures, and the number of cells was directly counted using a 20-μm-deep hemocytometer (Sunlead Glass) and BX51 Fluorescence Microscope (Olympus).

To construct Bd-USDA 110 tagged with GFP and Bj-USDA 6 tagged with DsRed, pRJPaph-gfp (Ledermann *et al.*, 2015) and pBjGroEL4::DsRed2 (Hayashi *et al.*, 2014) plasmids were transferred by triparental mating using pRK2013 as a helper plasmid (Figurski and Helinski, 1979). Single recombination events, indicating the integration of pRJPaph-gfp into the Bd-USDA 110 chromosome or pBjGroEL4::DsRed2 into the Bj-USDA 6 chromosome, were selected based on their acquisition of resistance to Tc or Sp/Sm, respectively. Single recombinant strains were further confirmed using the BX51 Fluorescence Microscope (Olympus).

In immunoblotting and heme-staining analyses, precultured *Bradyrhizobium* cells were inoculated into 500-mL flasks containing 100‍ ‍mL of HMNN (OD_660_≈0.03) and incubated under microaerobic or anaerobic conditions at 30°C, with reciprocal shaking at 100 rpm. Soluble and membrane fractions were prepared as described previously (Fischer *et al.*, 2001; Torres *et al.*, 2014a). Soluble fractions were concentrated using Amicon Ultra-15 centrifugal filter units (Merck). In immunoblotting experiments, rabbit polyclonal antibodies were raised against the synthetic NapA peptide, CPLQKKATGAAKAND (GenScript Japan), which is common between the Nap of Bd-USDA 110 and Bj-USDA 6. Soluble fractions (10‍ ‍μg) were separated by SDS-PAGE (10% polyacrylamide) and transferred to P plus PVDF membranes (ATTO) using a HorizeBLOT 4M-R semi-dry transfer system (ATTO). Membranes were incubated with an anti-NapA antibody (1:10,000) at room temperature for 30‍ ‍min and then with HRP-conjugated anti-rabbit IgG (1:2,500; Promega) for 30‍ ‍min. Immunoreactive bands were detected using the chemiluminescent substrate, Promega^TM^ ECL Western Blotting Substrate. Regarding heme staining, membrane fractions (10‍ ‍μg) were separated by SDS-PAGE at 4°C (stacking phase at 5% acrylamide and resolving phase at 12% acrylamide), transferred to a 0.45 μm Hybond-P membrane (GE Healthcare), and stained with the Promega^TM^ ECL Western Blotting Substrate to detect heme-dependent activity by chemiluminescence (Vargas *et al.*, 1993). Blots were visualized using a Molecular Imager Gel Doc XR+ System with Image Lab software (Bio-Rad). Relative band intensities were calculated using ImageJ software (Schneider *et al.*, 2012). Experiments were performed with at least two independent biological replicates.

The alignment of the *napEDABC* genome regions of Bj-USDA 6 (accession number NC_017249) and Bd-USDA 110 (accession number NC_004463) was performed using Genome Matcher version 2.2 (Ohtsubo *et al.*, 2008). Amino acid alignment was conducted using MEGA version 7.0 and BoxShade version 3.21 (https://www.ch.embnet.org/software/BOX_form.html). A protein structural similarity analysis was performed using TM-align version 20170708 (Zhang and Skolnick, 2005).

The impaired activity of Nap in *B. japonicum* during anaerobic growth in the presence of NO_3_^–^ may rely on posttranscriptional effects that affect the structure, function, or level of Nap (Siqueira *et al.*, 2017). The *napEDABC* gene cluster of *B. diazoefficiens* encodes NapE, a membrane protein of 6.6‍ ‍kDa with unknown function; NapD (11.8‍ ‍kDa), a chaperone involved in the maturation of NapA; NapA (94.5‍ ‍kDa), the catalytic subunit that contains a Mobis-MGD active site and [4Fe4S] cluster; and NapB (16.9‍ ‍kDa), the electron transfer subunit, which is a diheme cytochrome *c*_552_. NapA and NapB form the heterodimeric subunit NapAB, which is present in the periplasm. NapC (25‍ ‍kDa) is a membrane-anchored, tetraheme, *c*-type cytochrome that is responsible for the transfer of electrons from the quinol pool to the NapAB complex (Zumft, 1997; Delgado *et al.*, 2003). A sequence comparison of the *napEDABC* gene cluster of Bd-USDA 110 and Bj-USDA 6 showed high identity (>85%) at the nucleotide level (Fig. S1). The amino acid sequences of Bj-USDA 6 NapEDABC were conserved, exhibiting 89–97% identity with Bd-USDA 110 (Fig. S1). In NapA of Bj-USDA 6, the twin arginine motif, the residues involved in the binding of the Mobis-MGD cofactor, and the [4Fe4S] cluster were conserved, except in some cases in which there was a substitution with a similar residue. In Bj-USDA 6 NapB, both the cytochrome *c*-binding sites and motif for translocation via the general secretory pathway were conserved. The structural similarity of the Bj-USDA 6 and Bd-USDA 110 Nap proteins was high, with a TM-score of 0.81, which indicated the same type of folding (Fig. S1). These results suggested that the structure and function of Nap were conserved in Bj-USDA 6.

We compared the amount of Nap in Bj-USDA 6 and Bd-USDA 110 cells incubated under microaerobic and anaerobic conditions in the presence of NO_3_^–^. An immunoblotting analysis of soluble fractions revealed a bradyrhizobial NapA protein band at ≈100‍ ‍kDa (Fig. 1A), which was consistent with the expected molecular weight of 94.5‍ ‍kDa (Delgado *et al.*, 2003). This was further confirmed by the absence of this band in soluble fractions of the *B. diazoefficiens* Δ*napA* mutant (Fig. 1A). The intensity of the NapA band was weaker in Bj-USDA 6 than in Bd-USDA 110, in cells incubated microaerobically (≈0.57 times; Fig. 1A and Table S1), and particularly in cells incubated anaerobically (≈0.33 times; Fig. 1A and Table S1). This result suggested that Bj-USDA 6 produced less NapA than Bd-USDA 110 under low-oxygen conditions (*i.e.* ≤2% O_2_) in the presence of NO_3_^–^. Additionally, we detected NapC in the membrane fractions of bradyrhizobial cells by heme staining; the staining of heme covalently bound to *c*-type cytochromes (Vargas *et al.*, 1993). Bj-USDA 6 and Bd-USDA 110 showed the typical profile of five stained bands previously identified in membrane fractions of Bd-USDA 110 (Fig. 1B) (Preisig *et al.*, 1993; Mesa *et al.*, 2002; Delgado *et al.*, 2003; Bueno *et al.*, 2008; Torres *et al.*, 2014a). The 32-kDa and 28-kDa bands, almost co-migrating, corresponded to the FixP and FixO proteins, respectively, of *cbb*_3_-type high affinity cytochrome oxidase, encoded by the *fixNOQP* operon (Preisig *et al.*, 1993, 1996); the 25-kDa band corresponded to NapC (Delgado *et al.*, 2003); the 20-kDa band corresponded to cytochrome CycM (Bott *et al.*, 1991); and the 16-kDa band corresponded to the NorC subunit of Nor (Mesa *et al.*, 2002). We confirmed the identities of bands by analyzing soluble fractions of the *B. diazoefficiens* Δ*napA* mutant under microaerobic conditions; bands correspondig to NapC and NorC were absent (Fig. 1B), as described previously (Delgado *et al.*, 2003). Under anaerobic conditions, the intensity of the NapC band was ≈0.3-fold weaker in Bj-USDA 6 than in Bd-USDA 110 (Fig. 1B and Table S1). Thus, immunoblotting and heme-staining results indicated that Bj-USDA 6 produced lower levels of Nap under low-oxygen conditions in the presence of NO_3_^–^. Therefore, impaired Nap activity in *B. japonicum* during NO_3_^–^-dependent anaerobic growth, which was reported previously (Siqueira *et al.*, 2017), may be caused by a low level of Nap.

Based on the results of heme staining, the amount of NorC was higher in Bj-USDA 6 than in Bd-USDA 110 under microaerobic and anaerobic conditions (≈1.3- and 1.6-fold, respectively) in the presence of NO_3_^–^ (Fig. 1B and Table S1). This result was supported by a previous finding showing that *norB* transcript levels were higher in Bj-USDA 6 than in Bd-USDA 110 under anaerobic NO_3_^–^-dependent growth (Siqueira *et al.*, 2017). Heme staining also revealed that besides NapC, FixP and FixO were the only other cytochromes affected in Bj-USDA 6 under low-oxygen conditions; the amounts of FixP and FixO were lower in Bj-USDA 6 than in Bd-USDA 110 by ≈0.45-fold under microaerobic conditions and ≈0.8-fold under anaerobic conditions (Fig. 1B and Table S1). This result suggested that *cbb*_3_ oxidase is a critical enzyme for the adaptation process to NO_3_^–^ respiration under low-oxygen conditions in *Bradyrhizobium*. Accordingly, the maximal expression of Nap does not appear to occur until the oxygen concentration becomes very low, and this is only observed after *cbb*_3_ oxidase has consumed the oxygen present in the growth medium (Bueno *et al.*, 2008). Since the transcription of *nap* genes appeared to be unaffected in *B. japonicum* cells grown anaerobically in the presence of NO_3_^–^ (Siqueira *et al.*, 2017), the remaining oxygen in the growth medium may affect the amount of the Nap protein at the posttranscriptional level by inhibiting the translation of *nap* messenger RNA or inducing the degradation of Nap proteins.

The mechanisms responsible for low levels of *cbb*_3_ oxidase in *B. japonicum* under low-oxygen conditions in the presence of NO_3_^–^ may be related to *B. japonicum* being unable to make an effective switch to denitrification in the absence of oxygen, similar to other bacteria (Aida *et al.*, 1986; Bergaust *et al.*, 2011; Torres *et al.*, 2014b; Siqueira *et al.*, 2017). We tested the NO_3_^–^-dependent anaerobic growth of bradyrhizobial cells preincubated microaerobically, instead of aerobically as previously reported (Siqueira *et al.*, 2017). The microaerobic preincubation resulted in a reduced growth rate of Bj-USDA 6, to a similar extent as the aerobic preincubation (Fig. S2), indicating the absence of a significant difference between a rapid (aerobic preincubation) and gradual (microaerobic preincubation) transition from aerobiosis to anaerobiosis. However, further studies are needed to elucidate the mechanisms responsible for the changes observed in *cbb*_3_ oxidase levels in *B. japonicum* under denitrifying conditions.

Our previous findings prompted the hypothesis that *B. japonicum* may be less competitive than *B. diazoefficiens* due to energy depletion under anaerobic denitrifying growth (Siqueira *et al.*, 2017). We examined the competitive growth of Bd-USDA 110 and Bj-USDA 6 tagged with GFP and DsRed proteins, respectively. The tagged strains were tested to exclude the possible effects of tagging on growth rates (Fig. S3). Under microaerobic and anaerobic conditions, in the presence of NO_3_^–^, the growth of DsRed-tagged Bj-USDA 6 was lower than that of GFP-tagged Bd-USDA 110, whereas the aerobic growth rates of these two species were similar (Fig. 2). This result indicated that Bd-USDA 110 is more competitive than Bj-USDA 6 at low-oxygen levels in the presence of NO_3_^–^. In *Bradyrhizobium*, growth at low-oxygen levels in the presence of NO_3_^–^ is supported by both *cbb*_3_ oxidase, which is active during free-living microaerobic growth and in N_2_-fixing bacteroids, and the denitrification pathway (Preisig *et al.*, 1993, 1996; Bueno *et al.*, 2008). The weaker competitiveness of *B. japonicum* USDA 6 under low-oxygen conditions in the presence of NO_3_^–^ may correlate with low levels of Nap (Fig. 1 and Table S1).

In conclusion, the present study demonstrated that the level of Nap under low-oxygen conditions in the presence of NO_3_^–^ was lower in *B. japonicum* USDA 6 than in *B. diazoefficiens* USDA 110 and suggests that the capacity to maintain a sufficient quantity of Nap is an advantage under denitrifying conditions. The lower levels of Nap in *B. japonicum* USDA 6 presumably resulted in a competitive disadvantage against *B. diazoefficiens* USDA 110 due to energy depletion during denitrifying growth. Ecologically, this advantage of *B. diazoefficiens* may be an important factor that influences the predominance of *B. diazoefficiens* over *B. japonicum* in soils exposed to low-oxygen conditions (Shiina *et al.*, 2014).

## Citation

Siqueira, A. F., Sugawara, M., Arashida, H., Minamisawa, K., and Sánchez, C. (2020) Levels of Periplasmic Nitrate Reductase during Denitrification are Lower in *Bradyrhizobium japonicum* than in *Bradyrhizobium diazoefficiens*. *Microbes Environ ***35**: ME19129.

https://doi.org/10.1264/jsme2.ME19129

## Supplementary Material

Supplementary Material

## Figures and Tables

**Fig. 1.**
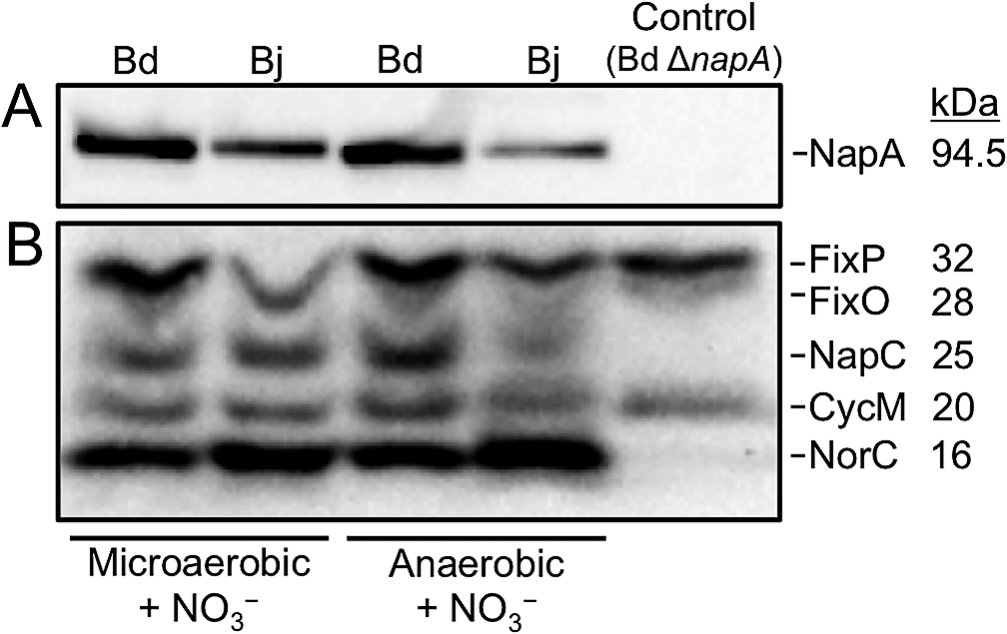
Detection of Nap in *Bradyrhizobium diazoefficiens* USDA 110 (Bd) and *Bradyrhizobium japonicum* USDA 6 (Bj) in HMMN medium under microaerobic and anaerobic conditions. (A) Immunoblotting analysis of NapA in soluble fractions (10‍ ‍μg). (B) Heme staining of membrane fractions (10‍ ‍μg). In panels A and B, the *B. diazoefficiens* USDA 110 Δ*napA* mutant grown in HMMN medium under microaerobic conditions is shown as the control.

**Fig. 2.**
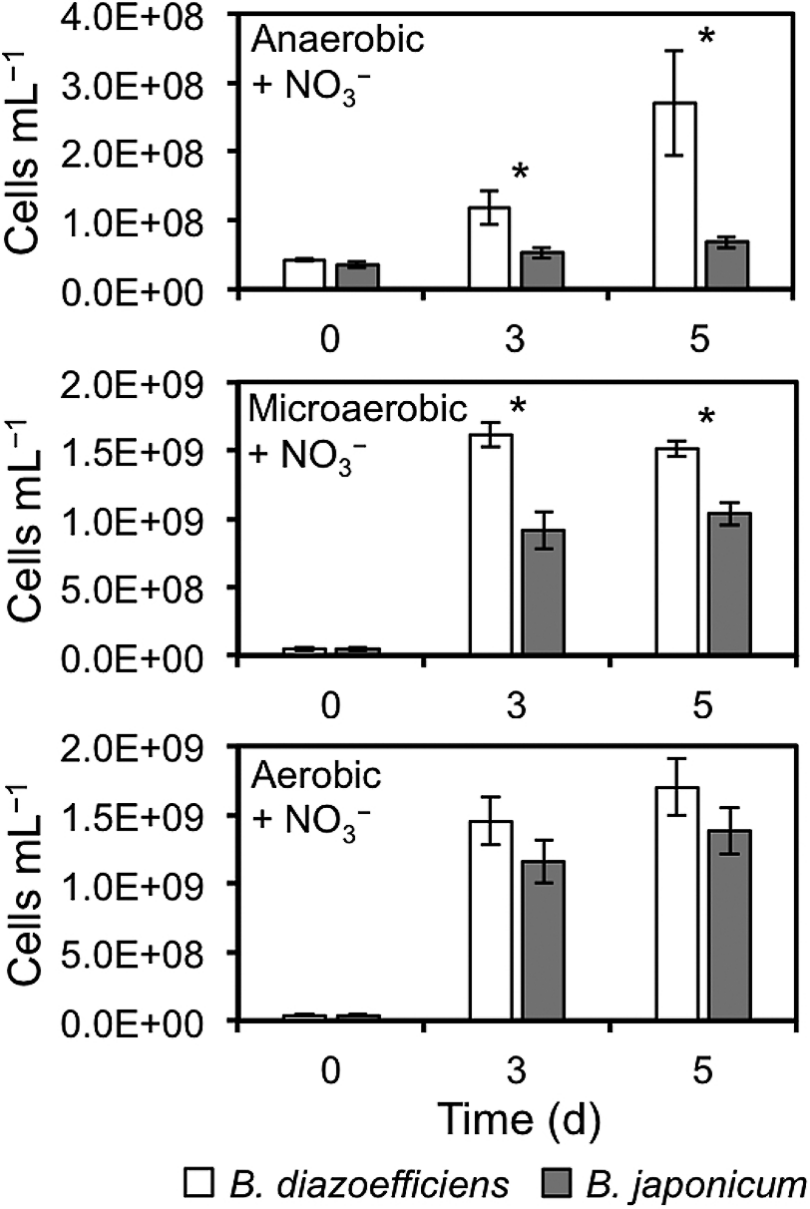
Average number of cells in co-cultures (1:1) of *Bradyrhizobium diazoefficiens* USDA 110 tagged with GFP (white bars) and *Bradyrhizobium japonicum* USDA 6 tagged with DsRed (grey bars) under anaerobic, microaerobic, and aerobic conditions in HMMN medium. Error bars indicate SE. * Values significantly different between USDA 110 and USDA 6 (*t*-test, *P*<0.05; *n*=3).
