# Supplementary material for: Levels of Periplasmic Nitrate Reductase during Denitrification are Lower in Bradyrhizobium japonicum than in Bradyrhizobium diazoefficiens
Source: Microbes Environ. 2020 Jun 16;35(3):ME19129. doi: 10.1264/jsme2.ME19129 (PMC7511789; doi:10.1264/jsme2.ME19129)
Supplement: Supplementary file 1 — Supplementary Material [file 35_19129_s1.pdf]

Supporting information

Levels of periplasmic nitrate reductase during denitrification are lower in *Bradyrhizobium japonicum* than in *Bradyrhizobium diazoefficiens*

Arthur Fernandes Siqueira[1], Masayuki Sugawara[1], Haruka Arashida[1], Kiwamu Minamisawa[1], Cristina Sánchez[1]*

[1] Graduate School of Life Sciences, Tohoku University, 2-1-1 Katahira, Aoba-ku, Sendai 980-8577, Japan

Running head: Low amount of Nap in *B. japonicum*

*Corresponding author:

E-mail: cristina.sago@gmail.com; Tel: +34–697–821–861; Fax: +81–22–217–5684

Table S1. Values of peak area, percentage of intensity (%) and relative band intensity (RI) of bands detected by heme-*c* staining (FixP/O, NapC, CycM, and NorC) and immunoblotting (NapA).

Protein	Microaerobiosis					Anaerobiosis					Control	
	USDA 110		USDA 6		RI[a,b]	USDA 110		USDA 6		RI[a,b]	USDA 110 Δ*napA*	
	Area[a]	%[a]	Area[a]	%[a]		Area[a]	%[a]	Area[a]	%[a]		Area[a]	%[a]
FixP/O	7668	39	3033	18	0.45	7075	34	4005	27	0.80	10838	70
**NapC**	2429	12	3556	21	**1.66**	3735	18	928	5	**0.30**	**ND**	**ND**
CycM	2015	10	2073	12	1.17	2595	12	1719	10	0.79	4625	30
NorC	7381	38	8488	49	1.31	7716	37	10261	58	1.59	ND	ND
**NapA**	12477	35	7111	20	**0.57**	11968	34	3900	11	**0.33**	**ND**	**ND**

[a] Values calculated using ImageJ (https://imagej.nih.gov/ij/).
[b] Relative band intensities of USDA 6 to those of USDA 110.
ND, not detected.

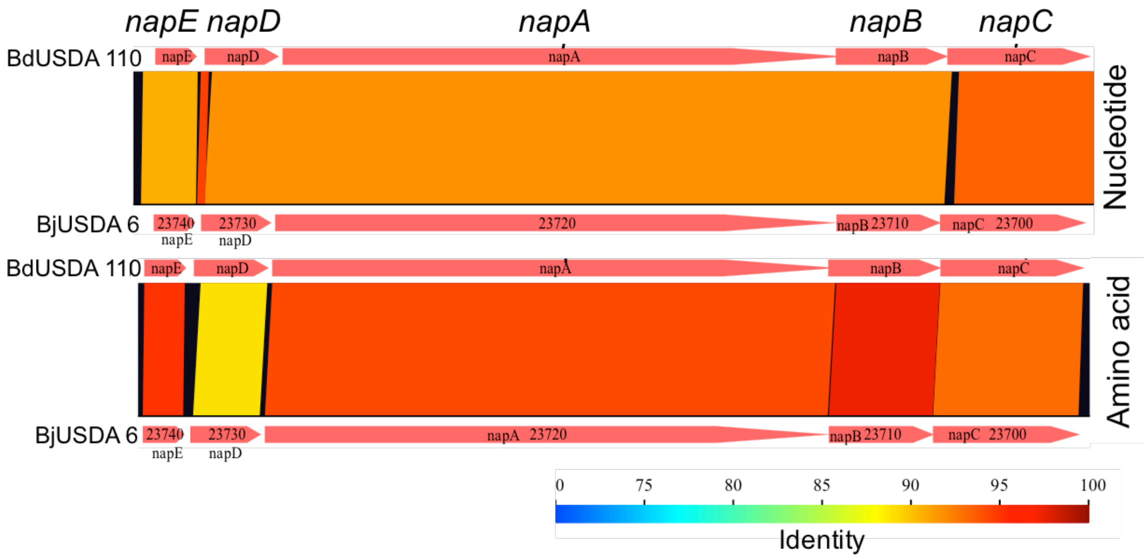

Target	Identity (%)		Protein structural similarity (TM-score)
	Nucleotide	Amino acid	
napE	91.67%	96.08%	-
napD	89.40%	89.00%	0.97
napA	91.85%	94.38%	0.99
napB	93.59%	97.42%	1
napC	93.03%	93.09%	0.83

Figure S1. Alignment of periplasmic nitrate reductase (Nap) sequences of *Bradyrhizobium diazoefficiens* USDA 110 and *Bradyrhizobium japonicum* USDA 6 at nucleotide, amino acid, and protein structure levels. TM-score, template modeling score.

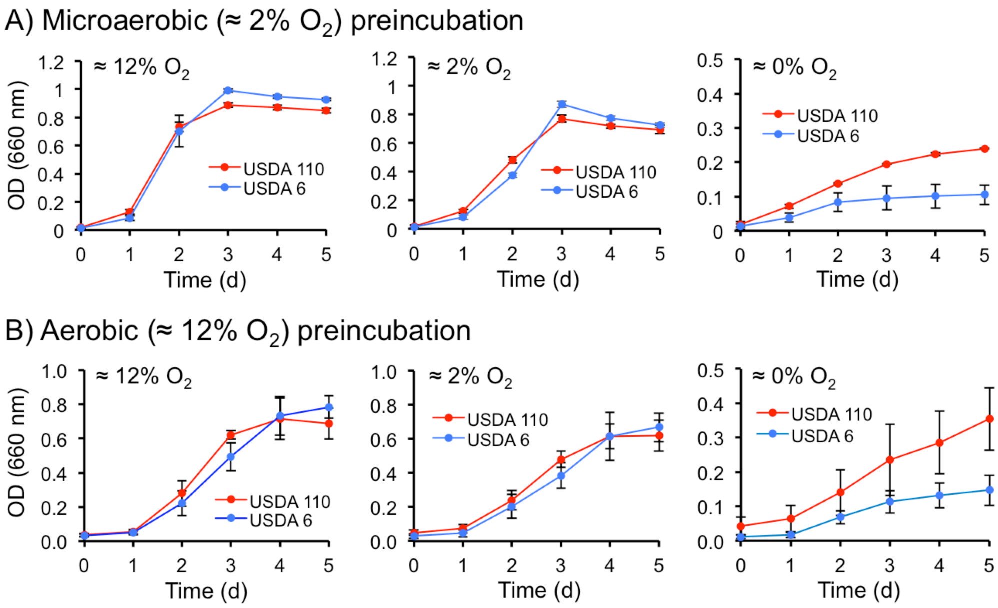

Figure S2. Aerobic (≈ 12% O_2), microaerobic (≈ 2% O_2), and anaerobic (≈ 0% O_2) growth of *B. diazoefficiens* USDA 110 (red) and *B. japonicum* USDA 6 (blue) in HMMN medium. Cells were preincubated microaerobically (A) or aerobically (B). Error bars indicate SE (n = 3).

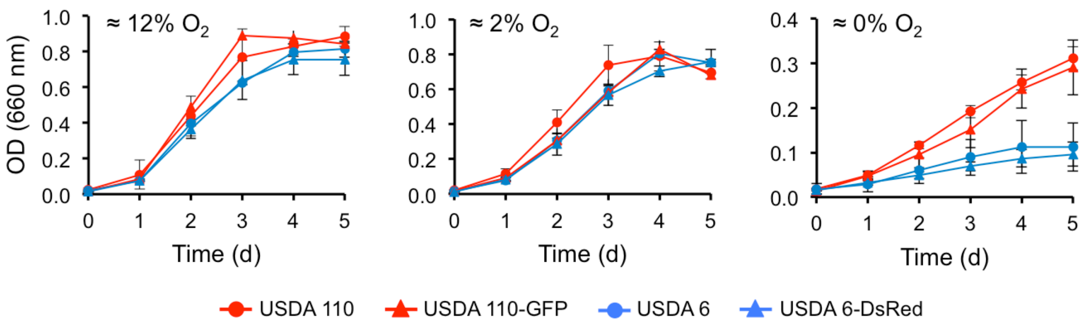

Figure S3. Aerobic (≈ 12% O_2), microaerobic (≈ 2% O_2), and anaerobic (≈ 0% O_2) growth of *B. diazoefficiens* USDA 110 (red circles) and USDA 110 tagged with GFP (red triangles), and *B. japonicum* USDA 6 (blue circles) and USDA 6 tagged with DsRed (blue triangles) in HMMN medium. Cells were preincubated aerobically. Error bars indicate SE (n = 3).
